# University of Buffalo Endowments

**Published:** 1904-08

**Authors:** 


A Monthly Review of Medicine and Surgery.

*EDITOR:*

WILLIAM WARREN POTTER, M. D.

All communications, whether of a literary or business nature, books for review and exchanges should be addressed to the editor: 284 FRANKLIN STREET, BUFFALO, N. Y.

VOL. XLIV.—LX. AUGUST, 1904. NO. I

### University of Buffalo Endowments.

WHEN the leader "Keep in touch with Buffalo" was published in the last issue of THE JOURNAL, it was for the purpose of arousing interest in the University of Buffalo among the alumni of the medical department. It was not expected that the task of bringing the question of an ardent and earnest support of the institution before the friends of the college would be an easy one, therefore a series of editorial articles on the same general subject was contemplated with the intention of hammering at it until either an active interest was awakened, or until it should become apparent that apathy was so dense that nothing could penetrate it.

It is gratifying, however, to state that within a very few days after THE JOURNAL had been placed in the mails, letters were received from some of the alumni and other friends of the University commending the stand taken and offering support. Personal visits to the editor of THE JOURNAL followed and so much genuinely unselfish interest has been shown on all sides, that we feel safe to assume the university will henceforth have at least the moral support of the alumni as a body. The exhibition of what might be called a pyrotechnic outburst of enthusiasm, will not suffice. The article referred to was written with the sole purpose of calling attention to apparent shortcomings. Its language was plain. Its silent text was covered by that epigram uttered by ex-Governor Black at a dinner given in his honor a few years ago at the Ellicott Club in Buffalo by Mr. Butler, of *The News*,—"He has no place on the heights who is satisfied with the plains." Its object was upward and onward.

The faculty of the medical department can no longer rest under the suspicion, even in the minds of the self-inflated or discontented, of inactivity. The council by its action at a recent meeting looking toward the establishment of a liberal arts department, has shown that it has not alone the interests of the university closely at heart, but the future educational welfare and reputation of the city of Buffalo as well. Yet the good-will and self-sacrificing endeavor of the gentlemen composing the council and those who have tirelessly worked as members of the medical faculty, are insufficient to bring the university before the world and place it in the position which by right of reputation and efficiency it deserves. Brains may make a university. Money is needed to support it. A man with money can always hire brains; but a man with brains, regrettable though it be, cannot always make money.

The University of Buffalo needs money. It has never in all its long and honorable career received one penny in endowment. Wealthy Buffalonians have given of their means to the church; they have given, with a swelling pride and a sense of work well done, liberally to charity at home and abroad; yet under their very eyes there has been for years struggling through the morass of adversity and hardship an educational institution of which the city should be proud, and which it should be considered a privilege and an honor to aid with a few of the dollars which they scatter with lavish hand. It is growing to be the fashion for rich men to give of their wealth during life. The motive for this innovation should not be questioned. It is well that it is so; it gives them a chance to see that they are doing good and aiding the cause of humanity.

When one scans closely the condition of the University of Buffalo and realises the pitiably small sum necessary to justify the establishment of a liberal arts' department, which necessarily means the enlargement and perfecting of the medical department, it seems as if there must be men in Buffalo sufficiently broad-minded and public-spirited to remove the only obstacle that lies in the way of a brilliant future. It is estimated that $25,000 a year would place the university in a position to take her place among well-equipped and well-qualified educational institutions. If there are not twenty-five men in Buffalo sufficiently interested to give $1,000 a year each for this purpose, then there may be fifty such who might be prevailed upon to give $500 a year. In the meantime, while these twenty-five or fifty men are being canvassed and impressed with the importance of endowment, there is a chance for the alumni to distinguish themselves by creating an annual fund for the medical department. No matter how little it may be, if each alumnus would pledge himself to give so much each year, a very considerable and useful sum would be realised. Think it over!

